# The dual targeting of insulin and insulin-like growth factor 1 receptor enhances the mTOR inhibitor-mediated antitumor efficacy in hepatocellular carcinoma

**DOI:** 10.18632/oncotarget.6836

**Published:** 2016-01-07

**Authors:** Claudia Pivonello, Mariarosaria Negri, Maria Cristina De Martino, Maria Napolitano, Cristina de Angelis, Donatella Paola Provvisiero, Gaia Cuomo, Renata Simona Auriemma, Chiara Simeoli, Francesco Izzo, Annamaria Colao, Leo J. Hofland, Rosario Pivonello

**Affiliations:** ^1^ Dipartimento di Medicina Clinica e Chirurgia, Università “Federico II” di Napoli, Naples, Italy; ^2^ IOS & Coleman Medicina Futura Medical Center, Centro Direzionale, Naples, Italy; ^3^ Immunology Oncology Unit, Istituto Nazionale per lo Studio e la Cura dei Tumori “Fondazione Giovanni Pascale” (IRCCS), Naples, Italy; ^4^ IRCCS Fondazione SDN, Istituto Nazionale per lo Studio e la Cura dei Tumori “Fondazione Giovanni Pascale” (IRCCS), Naples, Italy; ^5^ Hepatobiliary Surgery Unit, Istituto Nazionale per lo Studio e la Cura dei Tumori “Fondazione Giovanni Pascale” (IRCCS), Naples, Italy; ^6^ Department of Internal Medicine, Division of Endocrinology, Erasmus MC, Rotterdam, The Netherlands

**Keywords:** HCC, IGF1R, mTOR, OSI-906

## Abstract

Deregulation of mTOR and IGF pathways is frequent in hepatocellular carcinoma (HCC), thus mTOR and IGF1R represent suitable therapeutic targets in HCC. The aim of this study was to evaluate the effects of mTOR inhibitors (mTORi) and OSI-906, blocker of IGF1R/IR, on HCC cell proliferation, viability, migration and invasion, and alpha-fetoprotein (α-FP) secretion. In HepG2 and HuH-7 we evaluated, the expression of mTOR and IGF pathway components; the effects of Sirolimus, Everolimus, Temsirolimus and OSI-906 on cell proliferation; the effects of Sirolimus, OSI-906, and their combination, on cell secretion, proliferation, viability, cell cycle, apoptosis, invasion and migration. Moreover, intracellular mechanisms underlying these cell functions were evaluated in both cell lines. Our results show that HepG2 and HuH-7 present with the same mRNA expression profile with high levels of IGF2. OSI-906 inhibited cell proliferation at high concentration, while mTORi suppressed cell proliferation in a dose-time dependent manner in both cell lines. The co-treatment showed an additive inhibitory effect on cell proliferation and viability. This effect was not related to induction of apoptosis, but to G0/G1 phase block. Moreover, the co-treatment prevented the Sirolimus-induced AKT activation as escape mechanism. Both agents demonstrated to be differently effective in inhibiting α-FP secretion. Sirolimus, OSI-906, and their combination, blocked cell migration and invasion in HuH-7. These findings indicate that, co-targeting of IGF1R/IR and mTOR pathways could be a novel therapeutic approach in the management of HCC, in order to maximize antitumoral effect and to prevent the early development of resistance mechanisms.

## INTRODUCTION

Hepatocellular carcinoma (HCC) is the sixth most common malignancy worldwide and the third most frequent cause of global cancer-related mortality [1, 2]. If diagnosed at early stage, HCC can be cured by surgical tumor resection or liver transplantation [2]. However, only 10-20% of patients are candidates for curative surgery, and approximately 80-90% of patients are not eligible for surgery because of the extent of tumor, the tumor spread and the level of underlying liver dysfunction [1–4]. HCC has been shown to be chemoresistant to the most common chemotherapic compounds [2]. In this setting, targeted therapies could represent a new therapeutic option in HCC patients. In particular, sorafenib, a small molecule multi-tyrosine kinase inhibitor, has been demonstrated to improve survival, in terms of median overall survival, in patients with advanced HCC [2, 5], although approximately one-third of patients treated with sorafenib experience disease progression [6]. Therefore, novel treatment options are required in the management of HCC.

Hepatocarcinogenesis has been suggested to be a complex multistep process characterized by a broad spectrum of molecular abnormalities [7]. The study of the molecular mechanisms involved in HCC pathogenesis has highlighted the existence of several signaling pathways, which might be targetable with new therapies, representing new potential treatments in patients with advanced HCC. Particularly, an increased expression of insulin-like growth factor (IGF) 2 and IGF1 receptor (IGF1R) [8–11] and a deregulation of the phosphatidylinositol 3-kinase (PI3K)/AKT/mammalian target of rapamycin (mTOR) pathways [7, 12] have been reported in HCCs.

The IGF signaling pathway is a controlled endocrine system that is physiologically involved in the regulation of human development, energy balance, cell growth and development and it is biologically active in many neoplastic processes [11]. IGF1R is a tyrosine kinase receptor sharing approximately 84% amino acid homology with insulin receptor (IR). IGF1R, activated by IGF1 and IGF2 derived from endocrine, autocrine and paracrine sources, transduces signals to PI3K/AKT/mTOR and mitogen-activated protein kinase (MAPK) signaling pathways, *via* insulin receptor substrate 1 (IRS-1), mediating the regulation of cell growth, proliferation, motility, invasion and metastasis, in a variety of human malignancies [13]. Therefore, the IGF pathway is considered a potential target for antineoplastic therapy in several tumors, including HCC [13, 14].

mTOR is a serine/threonine protein kinase that controls cell growth in response to nutrients and growth factors and it has been found to be frequently deregulated in cancer. mTOR resides in two distinct complexes named mTOR complex 1 (mTORC1) and 2 (mTORC2); both complexes are activated by distinct upstream signals, regulate different biological processes and have different sensitivities to rapamycin, a macrolide ester produced by *Streptomyces hygroscopicus* [15–17]. Activation of the PI3K by growth factors results in the phosphorylation of AKT which consequently elicits mTORC1 activation, leading to the downstream phosphorylation of two main effectors: eukaryotic translation initiation factor 4E-binding protein 1 (4eBP1 (EIF4EBP1)) and ribosomal protein S6 kinase 1 (p70S6K (RPS6KB1)). Both 4eBP1 and p70S6K are regulators of mRNA translation and stimulate the synthesis of several proteins involved in cell proliferation [18]. The use of rapamycin (Sirolimus) and its analogues (mTOR inhibitors (mTORi)) in preclinical and clinical investigations has revealed that mTORC1 pathway is involved in a network of signaling cross-talk and feedback mechanisms and that mTORC1 inhibition results in loss of negative feedback loop and in the activation of AKT and MAPK pathway, reducing the effectiveness of mTORi in cancer treatment [19–21]. Therefore, a potential concomitant suppression of PI3K/AKT/mTOR and MAPK pathways, targeting IGF1R/IR and mTOR, can be highly detrimental for tumor growth.

The aims of the current study were: 1) to characterize the IGF and mTOR pathways, 2) to evaluate the effect of IGF1R/IR blocking, using the dual inhibitor OSI-906, and mTOR blocking, using several mTORi as single agents and 3) to evaluate wheter the combination of the two categories of compounds has an additive effect in the regulation of cell secretion, proliferation, migration, invasion and cell cycle in two HCC cell lines.

## RESULTS

### mTOR and IGF pathway components are expressed in HCC cell lines and HCC tissues

**Figure 1:**
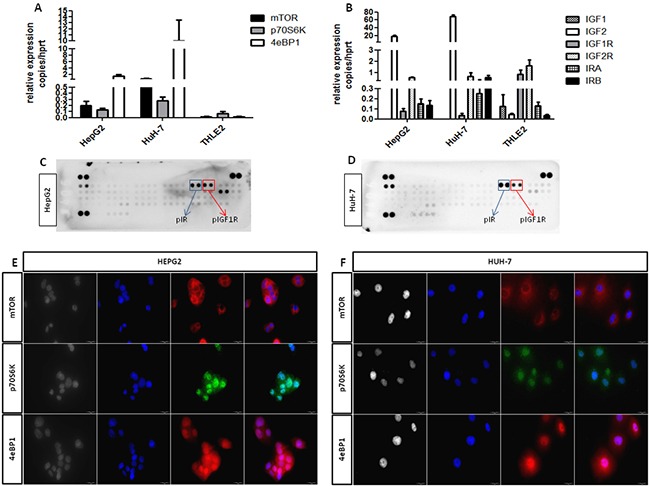
**A.** and **B.** mRNA relative expression of components of the mTOR and IGF pathways. **C.** and **D.** protein expression of phospho-IR and phospho-IGF1R in HepG2 and HuH-7 cells. **E.** and **F.** protein expression of mTOR components in HepG2 and HuH-7 cell lines. Black and white: contrast phase images. Blue: DAPI for nuclei staining. Green: FITC for p70S6K staining. Red: TRITC for mTOR and 4eBP1 staining.

In the attempt to define the role of mTOR and IGF pathways in HCC, RT-qPCR was performed to quantify the messenger expression level of mTOR, 4eBP1, p70S6K, IGF1, IGF2, IGF1R, IGF2R and IR (isoforms A and B), in THLE-2, HepG2 and HuH-7 cell lines, in normal liver and in HCC and peritumoral tissues. Both HCC cell lines presented the same messenger expression profile as shown in (Figure 1A and 1B). All mTOR and IGF pathway components were expressed in HCC tissues (Supplementary Figure S1 and Supplementary Figure S2) and in THLE-2, HepG2 and HuH7 cells (Figure 1A and 1B) except IGF1 that was surprisingly not detectable in both HCC cell lines. The messenger levels of mTOR and IGF pathway components of cell lines were compared to the normal hepatocytes (THLE-2) and are reported in Supplementary Table S1 as mean±S.E.M. mTOR components and activated forms of IGF1R and IR were expressed at protein levels in HepG2 and HuH-7 cells as shown in (Figure 1C, 1D, 1E and 1F). In both HCC cell lines, mTOR was essentially located at perinuclear and cytoplasmic level, whereas, p70S6K and 4eBP1 were sited in the nucleus and in the cytoplasm. In both HCC cell lines, IR and IGF1R resulted to be activated in basal condition.

### OSI-906 exerts a moderate antiproliferative effect in HCC cell lines

**Figure 2:**
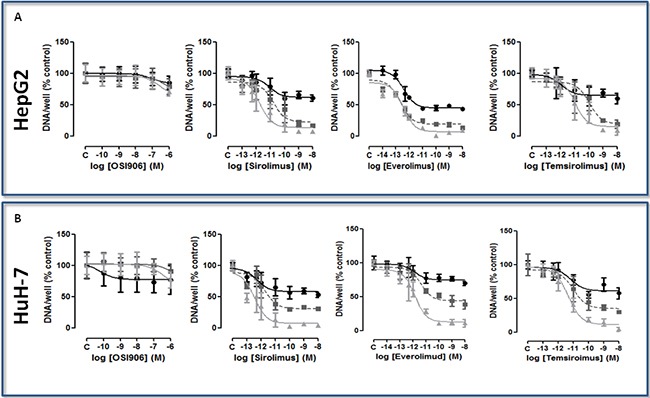
**A.** and **B.** Dose-time-dependent effect of the treatment with mTORi on cell proliferation, expressed as DNA content/well after 3 days (closed circle, black), 6 days (closed square, gray), and 9 days (closed triangle, light gray) of treatment, in HepG2 and HuH-7. Data are expressed as percentage of control and represent the mean ± S.D. of three independent experiments. Control is set as 100%.

In both HCC cell lines, the treatment with OSI-906 at serial concentrations between 10^−10^ and 10^−6^M did not induce a clear dose- and time-dependent inhibition of proliferation. In both cell lines, the maximum effect was reached only at the maximum dose (10^−6^M) after 9 days of treatment: (HepG2: 30% of inhibition, p<0.001; IC50>100nM); HuH-7: 24% of inhibition, p<0.001; IC50>100nM). Dose-time response curves for both cell lines are shown in (Figure 2A and 2B). The IC50 and the inhibition of cell proliferation after 3, 6 and 9 days of treatment in both cell lines are summarized in Supplementary Table S2. The value of inhibition is reported as percentage (p value vs control).

### mTORi induce dose-dependent and time-dependent antiproliferative effect in HCC cell lines

SIR, EVE and TEM significantly suppressed cell proliferation in a dose- and time-dependent manner (Figures 2A and 2B). In HepG2 all the mTORi significantly inhibited cell proliferation after 3, 6 and 9 days of treatment. In these cells, all the compounds showed the maximum effect after 9 days of treatment at the highest tested concentration (10^−8^M): the inhibitory effects of SIR, EVE and TEM were 93.16% (p<0.001), 91.13% (p<0.001) and 91.28% (p<0.001), compared to the control, respectively. In HuH-7 all the mTORi were able to significantly inhibit cell proliferation after 3, 6 and 9 days of treatment. The maximum effect was reached after 9 days of treatment at the highest tested concentration (10^−8^M): the inhibitory effects of SIR, EVE and TEM were 95.9% (p<0.001), 83.32% (p<0.001) and 95.83% (p<0.001), compared to the control, respectively. The IC50 and the inhibition of cell proliferation after 3, 6 and 9 days of treatment in both cell lines are summarized in Supplementary Table S2. The value of inhibition is reported as percentage (p value vs control).

### Combining Sirolimus and OSI-906 induces an additive inhibition of proliferation and cell viability in HCC cell lines

**Figure 3:**
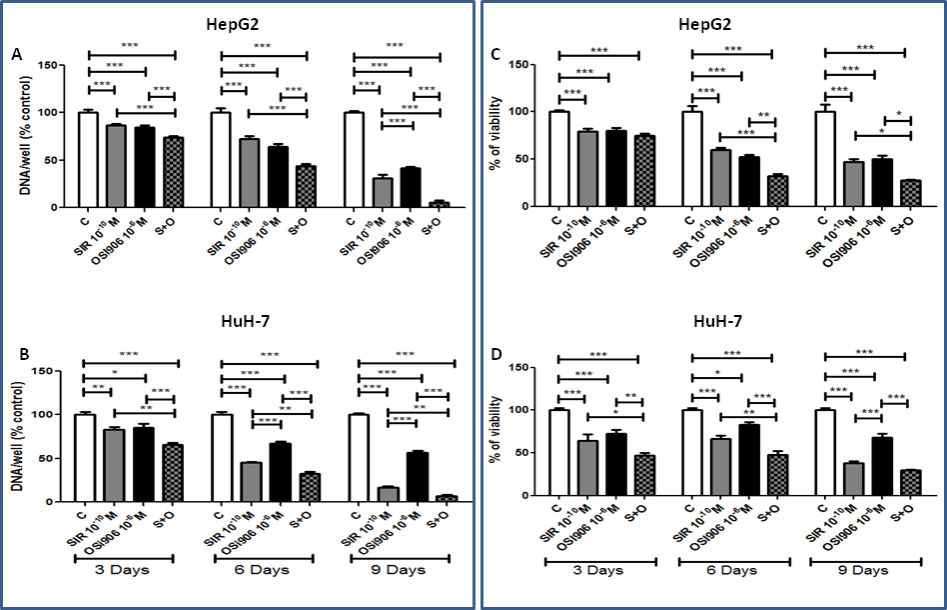
Antitumor effects of Sirolimus and OSI-906, alone and in combination, in both cell lines Since both drugs were diluted in DMSO, a double amount of DMSO was added to the control wells and the cells treated with the single agents were supplemented with an extra amount of DMSO. Data are expressed as percentage of control and represent the mean ± S.E.M. of at least three independent experiments for each cell line. Control is set as 100%. *P<0.05; **P<0.01; ***P<0.001. Drugs effects on **A.** HepG2 cell proliferation and on **C.** HepG2 cell viability, on **B.** HuH-7 cell proliferation and on **D.** HuH-7 cell viability.

In order to show an enhancement strengthening of the antineoplastic effect of OSI-906 and SIR, cells were treated with 10^−10^M SIR and 10^−6^M OSI-906, as single agents and in combination (S+O). After 3, 6 and 9 days of treatment, SIR and OSI-906 significantly suppressed cell proliferation and the drug combination induced an additive inhibition of cell proliferation in both cell lines (Figure 3A and 3B). In HepG2, S+O induced a 94.43% of inhibition after 9 days (p<0.001 *vs* control, p<0.001 *vs* SIR and p<0.001 *vs* OSI-906) (Figure 3A) of treatment. In HuH-7, S+O induced a 92.64% of inhibition after 9 days (p<0.001 *vs* control, p<0.01 *vs* SIR and p<0.001 *vs* OSI-906) (Figure 3B).

The combined treatment further inhibited HepG2 cell viability compared to single agents showing an additive effect after 6 and 9 days of treatment (72.85% of inhibition: p<0.001 *vs* control, p<0.05 *vs* SIR and p<0.05 *vs* OSI-906) (Figure 3C). The drug combination showed additive inhibition of HuH-7 cell viability after 3 and 6, with a maximum effect (70.29%) of inhibition after 9 days (p<0.001 *vs* control and p<0.001 *vs* OSI-906) (Figure 3D) in which the effect was not significant different from the effect of SIR.

### Sirolimus and OSI-906 regulate cell cycle profiles in HCC cell lines

**Figure 4:**
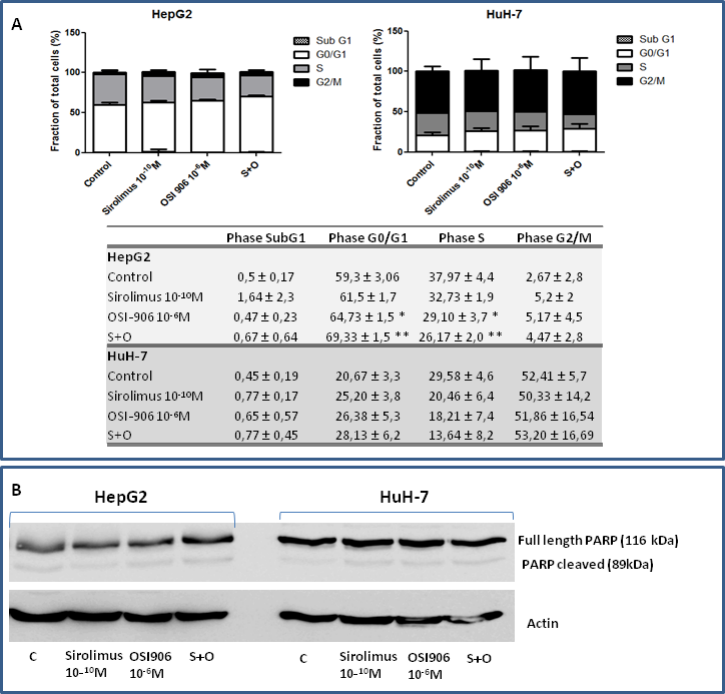
**A.** Analysis of cell cycle repartition by flow cytometry in both cell lines. Data are reported as mean ± S.E.M. of three independent experiments for each cell line. **B.** Effects of Sirolimus and OSI-906, alone and in combination, on PARP cleavage in HepG2 and HuH-7 cell lines.

To determine the effects of OSI-906 and SIR on cell cycle progression, HepG2 and HuH-7 cells were treated with drugs alone and in combination and analyzed for cell cycle phase. As shown in Figure 4A, in HepG2, 24hrs of combined treatment with SIR and OSI-906 resulted in an increased rate of arrested cells in G0/G1 phase, compared to each compound alone (OSI-906 *vs* control p<0.05; S+O *vs* control p<0.01; S+O *vs* OSI-906 p<0.05; S+O *vs* SIR p<0.01) and, accordingly, in a decreased percentage of cells in the S phase (OSI-906 *vs* control p<0.05; S+O *vs* control p<0.01). In HuH-7, 24hrs single and combined treatment with SIR and OSI-906 did not induce a significant increased rate of arrested cells in G0/G1 phase compared to the control. No increased rate of cells in subG1 phase was present in both cell lines treated with SIR and OSI-906, alone or in combination, indicating the absence of apoptotic cells, as confirmed by the lack of Poly(ADP-ribose) polymerase (PARP) cleavage (Figure 4B).

### Sirolimus and OSI-906 induce a reduction of α-FP secretion in HCC cell lines

**Figure 5:**
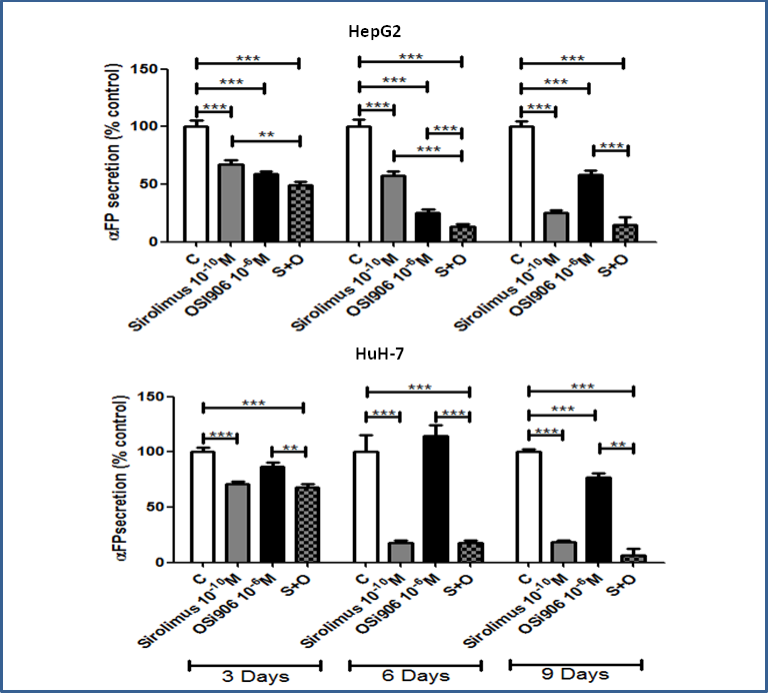
Antisecretive effects of Sirolimus and OSI-906, alone and in combination, after 3, 6 and 9 days of treatment in HepG2 and HuH-7 cell lines α-FP levels are expressed as percentage of control and represent the mean ± S.E.M. of three independent experiments for each cell line. Control is set as 100%. **P<0.01; ***P<0.001.

To test the effect of both drugs on cell secretion, α-FP secretion levels were measured in cell culture medium by CLIA. SIR and OSI-906 induced a significant inhibition of α-FP secretion, normalized to the amount of DNA in each well, in HepG2 and HuH-7, suggesting a direct effect of the two drugs on α-FP secretion. In HepG2, SIR 10^−10^M and OSI-906 10^−6^M induced a significant inhibition of α-FP secretion after 3, 6 and 9 days of treatment (Figure 5). SIR caused the maximum effect after 9 days of treatment (75%, p<0.001 *vs* control), while the maximum effect of OSI-906 was reached after 6 days of treatment (42,4%, p<0.001 *vs* control). The OSI-906 effect was partially lost after 9 days of treatment. The co-treatment induced the 87% of inhibition (p<0.001) after 6 days and this effect was still sustained after 9 days of treatment.

In HuH-7, SIR 10^−10^M induced the maximum inhibition after 6 days of treatment (82%, p<0.001 *vs* control) and this effect was maintained also after 9 days. OSI-906 10^−6^M inhibited α-FP secretion only after 9 days with a maximum inhibitory effect of 23% (p<0.001 *vs* control). The combined treatment reached the maximum inhibitory effect after 9 days (93%, p<0.001 *vs* control). Nevertheless, although this effect was greater than that obtained with the single treatments, it was not significantly different from the α-FP secretion inhibition induced by the single treatment with SIR (Figure 5).

### Sirolimus and OSI-906 suppress migration and invasion in HuH-7 cell line

**Figure 6:**
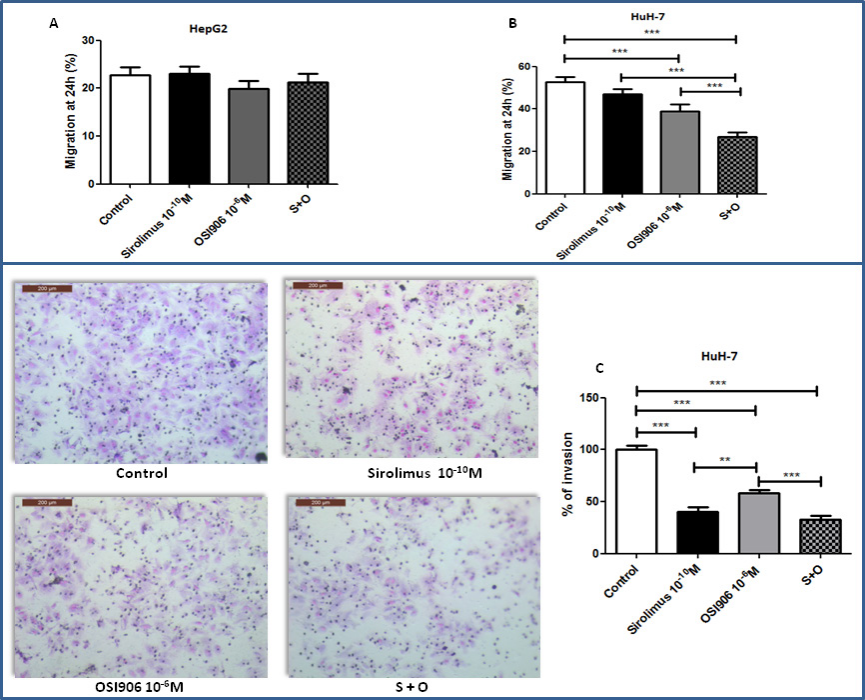
**A.** and **B.** effects of Sirolimus and OSI-906, alone and in combination, on cell migration after 24hrs of treatment, in HepG2 and HuH-7 cell lines. Data are reported as mean ± S.E.M. of three independent experiments for each cell line. Control is set as 100%. ***P<0.001. **C.** Effects of Sirolimus and OSI-906, alone and in combination, on cell invasion after 24hrs of treatment in HuH-7 cell line. Since both drugs were diluted in DMSO, a double amount of DMSO was added to the control wells and the cells treated with the single agents were supplemented with an extra amount of DMSO. **P<0.01; ***P<0.001.

At the experimental condition tested, the extent of migration of untreated HepG2 cells was very low and neither OSI-906 nor SIR further suppressed HepG2 cell migration (Figure 6A). In HuH-7, SIR did not inhibit cell migration (Figure 6B), while OSI-906 (p<0.001 *vs* control) and drugs combination (p<0.001 *vs* control) significantly decreased HuH-7 cells migration. Interestingly, in HuH-7, the combined treatment showed an inhibition of 53% compared to the control and an additive effect in driving the inhibition of cell migration compared to single agents (p<0.001 *vs* control, SIR and OSI-906). Matrigel invasion assay was performed to investigate cell invasion capability. At the experimental condition tested, HepG2 did not show invasion competence (Supplementary Figure S3). In HuH-7, SIR and OSI-906 caused a remarkable decrement of cell invasion, corresponding to 60% and 47%, respectively, (p<0.001 *vs* control for both drugs) while the combined treatment did not show additive inhibition compared to treatment with each compound as a single agent (Figure 6C).

### Combination of Sirolimus and OSI-906 regulates AKT/mTOR/p70S6K and ERK pathways in HCC cell lines

**Figure 7:**
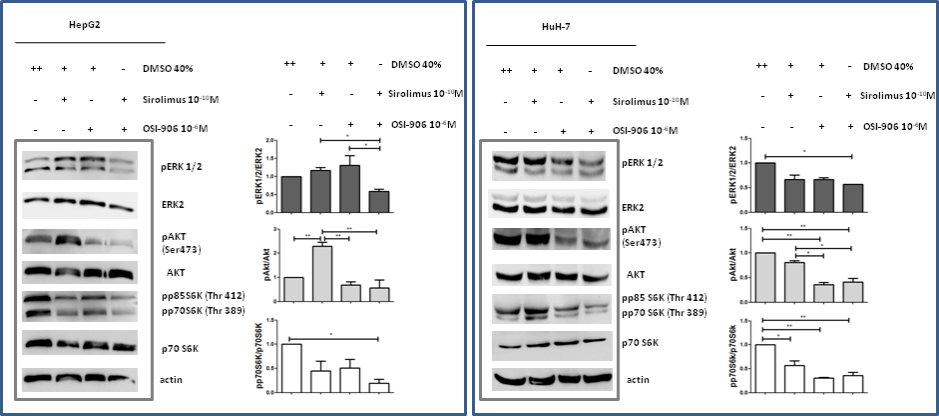
Effects of Sirolimus and OSI-906, alone and in combination, on AKT, p70S6K and ERK signalings after 30′ of treatment with drugs, in HepG2 and HuH-7 cell lines The blot in the figure is representative of at least two independent experiments for each cell line. Graphs represent the mean of at least two independent experiments for each cell line. *P<0.05; **P<0.01.

To investigate whether the cytostatic effects of OSI-906 and SIR were correlated with changes in intracellular components belonging to both mTOR and IGF pathways, AKT/mTOR/p70S6K and ERK signaling have been investigated. Whole-cell extracts in the presence of SIR and OSI-906, as single agents and in combination, were analyzed by Western blot. In HepG2, SIR did not induce a significant decrement of serum-induced p70S6K phosphorylation (Ser389), nor changed ERK1/2 (p42/p44) phosphorylation, but caused a significant induction of AKT phosphorylation (Ser473) (p<0.01 vs control). When used alone, OSI-906 did not caused any changes in the p70S6K, ERK1/2 and AKT phosphorylation compared to control. When OSI-906 was combined with SIR, serum-induced p70S6K phosphorylation was markedly inhibited compared to control (p<0.05) and further reduced compared to the effects of each drug used alone. Moreover, the activation of AKT and ERK1/2 was prevented by the combined treatment (Figure 7).

In HuH-7, SIR induced a significant decrement of serum-induced p70S6K phosphorylation (Ser389) (p<0.05) but no changes were observed in AKT and ERK1/2 phosphorylation. The treatment with OSI-906 induced a significant inhibition of p70S6K and AKT phosphorylation (p<0.01 *vs* control) but no change of serum-induced ERK1/2 phosphorylation. After combined treatment no additive effect on AKT and p70S6K dephosphorylation was reported, but the combination of both agents resulted in a stronger dephosphorylation of ERK1/2 (p<0.05 *vs* control) (Figure 7).

## DISCUSSION

HCC represents a chemoresistant aggressive type of cancer with generally poor prognosis [2, 4, 22]. Sorafenib is the only systemic therapy that has been demonstrated to prolong median survival and the time to progression in patients with advanced HCC [2, 5]. However, the overall outcomes are far from being satisfactory, firstly because of the genetic heterogeneity of HCC that can cause primary resistance to sorafenib and secondly because long-term exposure to sorafenib often results in acquired resistance of tumor cells to drug with consequent disease progression [6]. Therefore, new therapeutic strategies for HCC are needed. More effective systemic treatment options may include the combination of agents with additive/synergistic activity against HCC. mTORi have been used in the treatment of various solid tumors [23–27]. Despite their outstanding preclinical antitumor activity, mTORi, used as monotherapy in the management of HCC, have not confirmed the anticipated efficacy [28, 29]. This is probably related to the activation of feedback compensatory loops leading to tumor cell resistance like the up-regulation of IRS-1 protein levels and the induction of IRS-1 phosphorylation [19], the activation of both Akt phosphorylation via mTORC2 [30–32] and MAPK pathway *via* S6K/PI3K/Ras pathway [20]. These mechanisms may counteract the anticancer efficacy and may explain the variable response rates, demonstrated in different tumor types, when treated with mTORi.

In this study, it has been hypothesized that the combination of a dual blocker of IGF1R/IR, OSI-906, with the mTORi SIR, would potentiate the antitumor effects of SIR used as a single agent in HCC cells, because down-regulating AKT and MAPK survival pathway upstream with OSI-906, the resistance to mTORi, induced by mechanisms of escape, can be overcome. Therefore, for the first time, the effects of treatments with an mTORi and OSI-906 in terms of cell proliferation, viability, secretion, migration and invasion have been analyzed in two of the most common used HCC cell lines. Firstly, the expression of mTOR and IGF pathway components has been investigated in two human HCC cell lines, in one normal hepatocytes cell line, and confirmed in normal liver tissue and in HCC tissues and related peritumoral tissues. Both cell lines expressed comparable messenger levels of mTOR components, with higher levels of 4eBP1and mTOR, compared to normal hepatocytes, and a similar profile for IGF pathway components, with higher IGF2 and IRB compared to normal hepatocytes. The mTOR components and the activated forms of IGF1R and IR expression have been also confirmed at protein levels in the investigated HCC cell lines. According to the literature, and confirmed by our study, HepG2 and HuH-7 do not express IGF1, but they express IGF1R and high levels of IGF2 [8]. This latter could lead to autocrine stimulation of growth and motility through IGF1R and/or IR [33–35], acting as an important driver for HCC cell growth. The messenger and protein expression of IR has also been investigated previously in these two cell lines [8, 36]. In particular, IR protein expression has been investigated by western blot and a qualitative evaluation of IR isoform expression was conducted by RT-PCR analysis [8, 36]. Here the expression of both IR isoforms was investigated by RT-qPCR and comparable messenger levels of IRA and IRB were found in HepG2 and in HuH-7.

The *in vitro* antiproliferative and antisecretory effects of the mTORi and of the dual IGF1R/IR blocker, OSI-906, were evaluated. All used mTORi were been tested for the first time for a long period (9 days). The mTORi treatment induced a significant inhibition of cell growth *in vitro*, in a dose-time dependent manner, in both cell lines. Conversely, OSI-906, after both short-term (3 days) and long-term (9 days) treatment, was much less effective than mTORi in inhibiting cell proliferation, demonstrating that blocking IGF1R and IR is not a sufficient condition to reduce cell proliferation. Nevertheless, in both cell lines, OSI-906 showed a modest antiproliferative effect at high concentration. These results are consistent with a previous published study [8], whose results demonstrated that IR and IGF2 expression levels are positively correlated with sensitivity to OSI-906. The decision to continue the experiments by combining OSI-906 with SIR in particular, among all the mTORi, was based on several observations. First of all, SIR is the first mTOR allosteric inhibitor; EVE and TEM are synthetic rapamycin analog compounds, also known as “rapalogs”. Moreover, SIR and OSI-906 have shown antitumor activity, as single agents, in preclinical studies in HCC [8, 37–39]. Secondly, PI3K/AKT/mTOR and MAPK pathways are critical for cell proliferation, survival and resistance to apoptosis and both SIR and OSI-906 interact with these pathways [8, 31], which are known to be deregulated in HCC. Therefore, we choose to combine SIR with OSI-906 at concentrations of 10^−10^ M and 10^−6^M respectively, taking into account that these concentrations are lower than the maximum concentration achieved in blood, in patients with solid tumors enrolled in Phase I/II clinical trials [40, 41].

The combined treatment with SIR and OSI-906 showed an additive antiproliferative effect, in terms of both proliferation and viability, compared to treatment with both agents used alone. The analysis of the mechanisms underlying these antiproliferative effects revealed that in HepG2 the combined treatment with SIR and OSI-906 did non induce a significant inhibition of ERK1/2 phosphorylation compared with the control, but caused a significant block of the ERK1/2 phosphorylation triggered by the single treatment with SIR and OSI-906. Moreover, the co-treatment significantly blocked the escape pathway mediated by AKT phosphorylation, which follows the inhibition of mTORC1. The inhibition of p70S6K activation induced by the co-treatment with SIR and OSI-906, was more pronounced than treatment with single agents, but not significantly different. In HuH-7, the co-treatment did not induce a stronger inhibition of ERK1/2 and p70S6K phosphorylation, compared to treatment with single agents. SIR was not able to block the AKT phosphorylation. Nevertheless, OSI-906 abrogated the escape pathway on AKT, induced by the treatment with SIR. Although SIR has been previously reported to induce cell apoptosis [42], G0/G1 phase arrest, and not apoptosis, appeared the predominant mechanism responsible for the observed antiproliferative effects of SIR and OSI-906 in single and combined treatment. It could be speculated that the arrest in G0/G1 phase, mediated by these drugs, is due to the trigger of senescence [43] or autophagy [44].

The most widely used onco-glycoprotein marker for detecting HCC is serum α-FP [45]. It has been demonstrated that α-FP has pleiotropic effects, affecting the processes of cell differentiation, cell proliferation and tumorigenesis [46, 47]. The results of the current study demonstrated that both drugs induced a significant reduction of α-FP secretion after 3, 6 and 9 days of treatment in HepG2, while SIR has a pivotal role in inhibiting α-FP secretion compared with OSI-906 in HuH-7, independently from the effect on cell proliferation. The mechanisms responsible for this effect still need to be clarified.

Cell migration and invasion are implicated in the pathophysiology of many diseases, including cancer [48]. Prior to and during metastatization, tumor cells undergo several morphological changes and acquire an increased motility potential. Cell migration and invasion are critical parameters in the formation of metastasis [48]. The inhibition of these two key-processes can block the metastatic process, based on neoplastic cells invasion into the surrounding tissue and intravasation into blood or lymphatic vessels [48]. In this study it was demonstrated for the first time that SIR and OSI-906 can arrest migration and invasion of HuH-7, and, furthermore, the combination of the two agents showed an additive inhibition of cell migration. HuH-7 is a selected HCC cell line with more mesenchymal characteristics compared to HepG2 cell line; indeed, it has been previously showed that HuH-7 cells express mesenchymal markers (Vimentin and Zeb1) contrarily to HepG2 cells, which mainly express epithelial markers (E-cadherin and ErbB3) [8]. The epithelial pattern of expression showed by HepG2 could suggest that these cells behave more like epithelial than mesenchymal cells, thus providing an explanation for the lacking invasion capability of this cell line.

In conclusion, although the use of mTORi and OSI-906 failed to provide beneficial clinical effects in HCC and other solid tumors when used as single agents, this *in vitro* study produces the evidences of a significant antitumor effect of the combined treatment with SIR and OSI-906 in human HCC cell lines. These results represent the rationale for the interest in the co-targeting of IGF1R/IR and mTOR pathways as a novel therapeutic approach in patients with HCC, in order to maximize the antitumor effect and to prevent the early development of resistance mechanisms.

## MATERIALS AND METHODS

### Study design

In this study, two HCC cell lines (HepG2 and HuH-7), one normal hepatocyte cell line (THLE-2) and seven HCC and peritumoral tissues were characterized for the mRNA expression of the most important components of the IGF and mTOR pathways. Moreover, in HCC cell lines, protein expression of mTOR components and p-IGF1R and p-IR has been detected as well. The effects of mTORi (Sirolimus (SIR), Everolimus (EVE), Temsirolimus (TEM)) and the dual inhibitor of IGF1R and IR, OSI-906, as single agents, were tested in dose- and time (3, 6 and 9 days)-dependent manner on cell proliferation. The serial doses used for these experiments ranged between 10^−14^ and 10^−8^M and between 10^−10^ and 10^−6^M for the mTORi and OSI-906, respectively. In order to explore the effects of mTORi in combination with OSI-906 in both human HCC cell lines, selected doses of SIR (10^−10^M) and OSI-906 (10^−6^M) were tested as single agents and in combination (S+O) at different incubation times according with the different experimental procedures, in particular after 3, 6 and 9 days of treatment on cell proliferation, viability and secretion, and after 24 hours of treatment on migration, invasion and cell cycle. In the experiments using the combination of two drugs, SIR was chosen among the different mTORi since it is considered the reference mTORi compound, and the selected doses used for the combined treatment were chosen as doses lower than blood maximal concentration (Cmax) reached in the target population of the pharmacokinetic studies reporting the therapeutic response of these agents [49, 50]. The times used for the different experiments were selected based on the appropriate times for each experimental methodology. In order to analyze the signalling mechanism underling the investigated processes, in both cell lines the effect of SIR plus OSI-906 on mTOR and MAPK components (AKT, ERK1/2, p70S6K phosphorilation) and on Poly(ADP-ribose) polymerase (PARP) cleavage were evaluated.

### Cell lines

THLE-2 cell line, derived from primary normal liver cells, was purchased from ATCC (American Type Culture Collection) and cultured in Williams E without fenol red with 10% FBS, 1 × 10^5^ U/L penicillin and streptomycin, cell maintenance cocktail B (Gibco) and 10mM Dexamethasone (Gibco). Two human HCC cell lines, HepG2 and HuH-7, were purchased from ECACC (European Collection of Cell Cultures) and Health Science Research Resources Bank (HSRRB), respectively. HepG2 cells, whose doubling time is around 48h, were cultured in RPMI 1640 medium with 10% of FBS, 1 × 10^5^ U/L penicillin and 2mmol/L L-glutamine, whereas HuH-7, whose doubling time is around 31h, were cultured in Dulbecco's Modified Eagle's Medium (DMEM) GlutaMax medium with 10% of FBS and 1 × 10^5^U/L penicillin and streptomycin. Cell lines were grown in a humidified condition in 5% CO_2_ at 37°C. HCC cell lines identity was confirmed by short tandem repeat profiling (LGC Standards Cell Line Authentication service).

### HCC human tissues

Seven frozen tissues and their peritumoral biospecimens have been supplied by Biobank of Istituto Nazionale per lo Studio e la Cura dei Tumori “Fondazione Giovanni Pascale” (IRCCS) of Naples. For the use of the clinical materials for research purposes, the Institutional Research Ethics Committee approved the study, and prior patient consent was obtained.

### Drugs and reagents

The mTORi, SIR, EVE and TEM, and the IGF1R/IR inhibitor, OSI-906, were purchased from LC Laboratories (Inc. Woburn, MA, USA). All compounds were dissolved in dimethylsulfoxide (DMSO) 100%, as recommended by the manufacturer, as stock solutions concentrated 10^−3^M and stored at −20°C. For each experiment, serial dilutions in DMSO 40% were freshly made prior to use.

### RNA isolation and quantitative RT-PCR

RT-qPCR was performed to quantify the messenger expression level of the most important mTOR pathway components (mTOR, 4eBP1 and p70S6K) and of IGF pathway components (IGF1, IGF2, IGF1R, IGF2R, and IR (isoforms A and B)) in THLE-2, HepG2, HuH-7 cell lines and in HCC and peritumoral tissues. The cells and the tissues were lysed on ice in a lysis binding buffer containing 100 mM Tris-HCl (pH 8.0), 500 mM LiCl, 10 mM EDTA (pH 8.0), 1% LiDS, 5 mM DTT, and 5 U/100 μl ribonuclease inhibitor (HT Biotechnology Ltd., Cambridge, UK). The mRNA was isolated from total RNA with the use of prewashed Dynabeads Oligo (dT)25 (Dynal AS, Oslo, Norway) for 10′ on ice. The beads were collected with a magnet and washed three times with 10 mM Tris HCl (pH 8.0), 0.15 M LiCl, 1 mM EDTA, 0.1% LiDS, and once with a similar buffer from which LiDS was omitted. The poly (A+) mRNA was eluted twice, for 2′ each time, in H_2_O (65°C) and 20 μl were used for cDNA synthesis in a Tris buffer (50 mM Tris–HCl (pH 8.3), 100 mM KCl, 4 mM DTT and 10 mM MgCl_2_) with 10 units RNase inhibitor, 2 units avian myeloblastosis virus Super Reverse Transcriptase, oligo dT (5 ng/μl) and 1 mM of each dNTPs in a final volume of 40 μl. This mix was incubated for 1 h at 42°C and the resulting cDNA was diluted fivefold in 160 μl sterile H_2_O. The cDNA was used for quantification of mRNA levels of all investigated genes. The total reaction volume (12.5 μl) consisted of 5 μl of cDNA and 7 μl of TaqMan Universal PCR Mastermix (Applied Biosystems, Branchburg, NJ, USA) with primers-probes. Primers and probes sequences and concentrations are shown in Supplementary Table S3. All primers and probes were purchased from Sigma-Aldrich. RT-qPCR was performed in 96-well optical plates with the TaqMan Gold nuclease assay (Perkin Elmer Corporation, Foster City, CA, USA) and the ABI Prism 7900 Sequence Detection System (Perkin Elmer, Groningen, The Netherlands), using the standard protocol. Briefly: after two initial heating steps at 50°C (2′) and 95°C (10′), samples were subjected to 40 cycles of denaturation at 95°C (15”) and annealing at 60°C (60”). All samples were assayed in duplicate. Values were normalized against the expression of the housekeeping gene phosphoribosyltransferase (HPRT). Results are expressed as mean of three different experiments. Dilution curves were constructed to calculate PCR efficiencies (E) for every primer–probe set [51]. Primer's efficiencies have been reported in Supplementary Table S3. The relative expression of target genes was calculated using the comparative threshold method, 2^−ΔCt^ [52] with efficiency correction of target and reference gene transcripts, as described previously [53]. To exclude genomic DNA contamination in RNA extracts, cDNA reactions were also performed without reverse transcriptase and amplified with each primer pair. To exclude contamination of the PCR mixtures, reactions were also performed in the absence of cDNA template, in parallel with cDNA samples.

### Immunofluorescence staining

The cells were fixed in 4% paraformaldehyde/1X PBS for 30 min at 4°C into culture-slides. Slides were washed thrice in 1X PBS for 5 min and placed in permeabilization buffer (Triton 0.25%/1X PBS) for 10 min. After subsequent thrice washing in 1X PBS for 5 min, slides were placed in blocking buffer (5% goat serum/Triton 0.1%/1X PBS) for 1h at RT. The cells were incubated with primary antibodies against mTOR (rabbit monoclonal Ab, clone 7C10, #2983 Cell Signalling, diluted 1:200), p70S6K (mouse monoclonal Ab, clone H-9, sc-8418 Santa Cruz, diluted 1:50), 4eBP1(rabbit monoclonal Ab, #9644 clone 53H11, Cell Signalling, diluted 1:200) in blocking buffer for 1h at RT. Slides were washed thrice in 0.1% Triton/PBS for 5 min and incubated 1h with rhodamine-conjugated secondary antibodies (ImmunoReagents, Inc., Raleigh, NC) diluted 1:1000 in blocking buffer for mTOR and 4eBP1 and with Fluorescein isothiocyanate (FITC)-conjugated secondary antibodies (Millipore, Temecula, California) diluited 1:1000 for p70S6K. A 4.6-Diamidino-2-phenylindole (DAPI) (Lonza Group Ltd, Basel, Switzerland) staining, diluted in PBS 1X 1:40000, was used to visualize the nuclei. Negative controls were performed, in which cells were incubated with secondary antibody with the omission of the primary antibody. Staining was visualized on an inverted microscope Olympus IX51 equipped for fluorescence and phase contrast microscopy (Olympus, Milan, Italy) and the images were captured at 40X magnification and acquired with Olimpus Digital Camera F-View II (Olympus, Milan, Italy).

### Phospho-receptor tyrosine kinase (RTK) array

Phospho-IGF1R and phosphor-IR were measured with Human Phospho-RTK Array Kit (#ARY001B) from R&D. HepG2 and HuH-7 cells were lysed and assays were carried out according to manufacturer's protocols.

### Cell proliferation assay

After trypsinization, HepG2 and HuH-7 cells were plated in 1 ml of complete culture medium in 24-well plates at different density, based on growth curves at 3, 6 and 9 days. For HepG2, 3.5 × 10^4^, 2.0 × 10^4^ and 5 × 10^3^ cells were plated for 3, 6 and 9 days, respectively. For HuH-7-1, 3.0 × 10^4^, 1.5 × 10^4^ and 5 × 10^3^ cells were plated for 3, 6 and 9 days, respectively. The plates were then placed in incubator in 5% CO_2_ at 37°C. After 24hrs, the test compounds were added to each well at different concentrations: SIR and TEM in a concentration range between 10^−13^ and 10^−8^M, EVE, that has been shown to be more effective than other mTORi in both cell lines, in a concentration range between 10^−14^ and 10^−8^M and OSI-906 in a concentration range between 10^−10^ and 10^−6^M. Controls were vehicle-treated. Plates were further incubated at 37°C and 5% CO_2_. In experiments with 6 and 9 days of treatment, medium was changed and compounds were fresly added every 3 days. After 3, 6 and 9 days of treatment, cells were harvested for DNA measurement. Also the combined effect of SIR (10^−10^M) and OSI-906 (10^−6^M) was evaluated after 3, 6 and 9 days of treatment. Measurement of total DNA content, representative for the number of cells, was performed using the bisbenzimide fluorescent dye (Hoechst 33258) (Boehring Diagnostics, La Jolla, CA), as previously described [54].

### Cell viability assay

After trypsinization, HepG2 and HuH7 cells were seeded in 96-well plates. Cells were plated in 100μL of culture medium at different density selected on the bases of previously performed growth curves at 3, 6 and 9 days. Particularly, 3 × 10^3^, 2 × 10^3^ and 1 × 10^3^ HepG2cells were plated for 3, 6 and 9 days, respectively. 2.5 × 10^3^, 1.5 × 10^3^ and 1 × 10^3^ HuH-7cells were plated for 3, 6 and 9 days respectively. Cells were exposed to a single and combined treatment with SIR (10^−10^M) and OSI-906 (10^−6^M). 10μl of MTT solution, concentrated 5 mg/ml in PBS, was added to 90 μl of medium in each well. The cells were then cultured for a further 1h. MTT was removed and 100 μL/well of solvent (isopropyl alcohol, 10% HCl) were added to the cells in constant stir for 15′ at room temperature. Absorbance was measured at 570 nm using a plate reader (Victor X4, Perkin Elmer, Italy). Vehicle-treated cells were used as control.

### Flow cytometry analysis of cell cycle

HepG2 and HuH-7 were plated at density of 1,5 × 10^5^ in 6-well plates in full medium. Cells were allowed to fully adhere for 24hrs, then, SIR (10^−10^M) and OSI-906 (10^−6^M) were added alone and/or in combination. Since both drugs were diluted in DMSO, a double amount of DMSO was added to the control wells and the cells treated with the single agents were supplemented with an extra amount of DMSO. After 24hrs of treatment, cells were harvested by trypsinization, washed with cold PBS, resuspended in PBS + EDTA, fixed in 96% ethanol and stored at −20°C overnight until the flow-cytometric assay was performed. Fixed cells were rinsed twice with PBS lacking calcium and magnesium and stained with 50μg/mL of propidium iodide (PI) (Sigma Aldrich) in the presence of Rnase A (10μg/mL) at room temperature in the dark for 1h. DNA content was determined by FACSCanto II flow cytometer (Becton Dickinson, San Jose, CA), with a 488nm Coherent laser. The channel FL2 was used to analyse 20,000 events. Data were acquired by FACSDiva software (Becton Dickinson, San Jose, CA) and analysis was performed by using Modfit version 2.0 software.

### Alpha-fetoprotein (α-FP) secretion

HepG2 and HuH-7 cells were cultured in 24-well plates and treated for 3, 6 and 9 days as previously described for cell proliferation. The amount of α-FP in the cell culture supernatant was analyzed by immuno-chemiluminescent assay (CLIA) (Liaison^®^, Diasorin, Torino, Italia).

### Cell migration analysis

The *in vitro scratch-wound healing assay* was used to study cell migration [55]. HepG2 and HuH-7 cells were seeded in poly-L-lysine pre-coated 12-well plates and were grown until 100% confluence. At this point, cells were exposed to 10^−10^M of SIR, 10^−6^M of OSI-906, and a combination of them, in culture medium containing a lower percentage of serum (1%), sufficient to prevent apoptosis or cell detachment and to minimize cell proliferation. After exposure, linear scratch-wounds were made in the cell monolayer using a p200 tip. Scratch wounds were then visualised using an inverted microscope, Zeiss Axiovert 40c, and pictures were captured by a digital camera, Canon power shot A640, at different time points: 0, 2hrs, 4hrs, 6hrs, 8hrs, and 24hrs. The areas of the scratch-wounds were analysed by Image J software (http://rsbweb.nih.gov/ij/). The rate of scratch closure was calculated by subtracting the remaining width of the scratch lines from the width of the baseline scratch (control) at consecutive time points. Each experiment was repeated at least three times. Four readings were made for each sample at each time point.

### Transwell invasion assay

Cell invasion was assessed using BioCoat Matrigel Invasion Chambers (BD) [56]. Matrigel Invasion Chambers were hydrated in the tissue culture incubator for 2hrs, by placing serum free medium on the bottom of the well and in the top of the chamber. After hydration of the Matrigel, the medium in the bottom of the well was replaced with 800 μl of medium containing 10% FBS. 5 × 10^4^ HepG2 and HuH-7 cells were plated in 200 μl of serum free medium in the top of the chamber and were exposed to 10^−10^M of SIR, 10^−6^M of OSI-906, and a combination of them. The invasion assay was carried out for 24hrs in the tissue culture incubator. Cells were fixed by replacing the culture medium on the bottom and top of the chamber with 3,7% formaldehyde dissolved in PBS for 15′ at room temperature and with methanol for 2′. After fixing the cells, the chambers were rinsed in PBS and stained with Giemsa for 10′. After washing the chambers several times by dipping the chambers in a large beaker filled with PBS, the cells (now blue in color) on top of the Matrigel membrane were removed with several Q-tips. All the cells that remained in the chamber were the ones that invaded the membrane, passed throughout the membrane, and ultimately reached the bottom side of the membrane. Invasiveness was determined by counting cells in five microscopic fields per well, using an inverted microscope equipped with 10X objective. The rate of inhibition (%) was calculated using the following formula: (1 – number of invading cells of the treatment group/number of invading cells of control group) × 100.

### Protein extraction and western blot analysis

HepG2 and HuH-7 cell lines were plated into culture dishes and grown to 70-80% confluence. After drug treatment (30′) of FBS-stimulated cells, cells were lysed in detergent buffer (1% NP-40, 10% Glycerol, 137mM NaCl, 20mM Tris pH7.6, 20mM NaF, 2μg/mL aprotinin, 2μg/mL leupeptin, 2μg/mL pepstatin, 200μM Na3VO4, 1mM PMSF) on ice for 30′. The homogenate was centrifuged for 15′, at 1200 x g and 4°C and the supernatant was collected and stored at −80°C until use. Protein concentrations were determined photometrically with a bicinchoninic acid (BCA) Protein Assay Kit (Thermo Scientific, USA). After protein heat-denaturation at 95°C for 10′, 40μg of total extracts were used for immunoblotting. Proteins from cell preparations were separated by 8% (according to protein's Molecular Weight detected) SDS-PAGE and then electroblotted onto a nitrocellulose membrane for 1h in a TransBlot Amersham apparatus. After a blocking treatment for 1h with 5% of milk, the nitrocellulose filters were probed with primary antibodies specific for ERK1/2 (sc-7383 Santa Cruz, Italy), ERK2 (sc-1647 Santa Cruz, Italy), pAKT (Ser 473) (#9271, Cell signalling, Italy), AKT (#9272, Cell signalling, Italy), pp70S6k (#9206, Cell signalling, Italy), p70S6k (sc-8418, Santa Cruz, Italy), PARP (#9542, Cell signalling, Italy) and β-actin (A4700; Sigma Aldrich, Italy) overnight; subsequently, filters were hybridated with peroxidise-conjugated secondary antibodies and immunoreactive bands were detected by ECL system. After chemiluminescent reaction, the blot was exposed to ImageQuant Las 4000 (GE Healthcare). The chemiluminescent signals of the appropriately sized bands were measured using the ImageQuant Las 4000 image system software.

### Statistical analysis

All the experiments were performed in quadruplicates and were replicated three times with the exception of western blot analysis that were replicated two times. All statistical analyses were performed using SPSS and GraphPad softwares. Non-linear regression (curve fit) analysis was used to identify the half-maximal inhibitory concentrations (IC50) for all used drugs. Differences between the treated groups were assessed by ANOVA, followed by a multiple comparative test (Newman-Keuls or Dunnett's correction).

## SUPPLEMENTARY MATERIALS FIGURES AND TABLES


