# Supplementary material for: The dual targeting of insulin and insulin-like growth factor 1 receptor enhances the mTOR inhibitor-mediated antitumor efficacy in hepatocellular carcinoma
Source: Oncotarget. 2016 Jan 7;7(9):9718–31. doi: 10.18632/oncotarget.6836 (PMC4891079; doi:10.18632/oncotarget.6836)
Supplement: Supplementary file 1 [file oncotarget-07-09718-s001.pdf]

SUPPLEMENTARY FIGURES AND TABLES

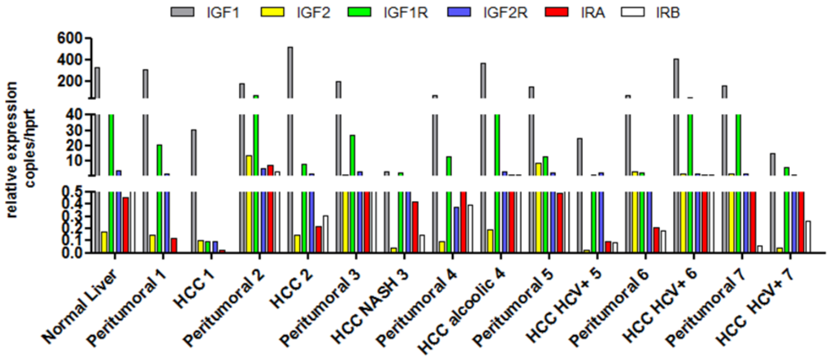

Supplementary Figure S1: mRNA relative expression of components of the IGF pathway in normal liver, HCCs and their peritumoral tissues.

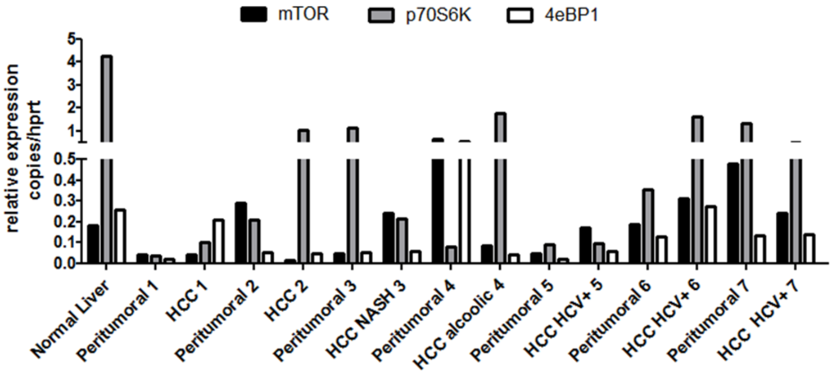

Supplementary Figure S2: mRNA relative expression of components of the mTOR pathway in normal liver, HCCs and their peritumoral tissues.

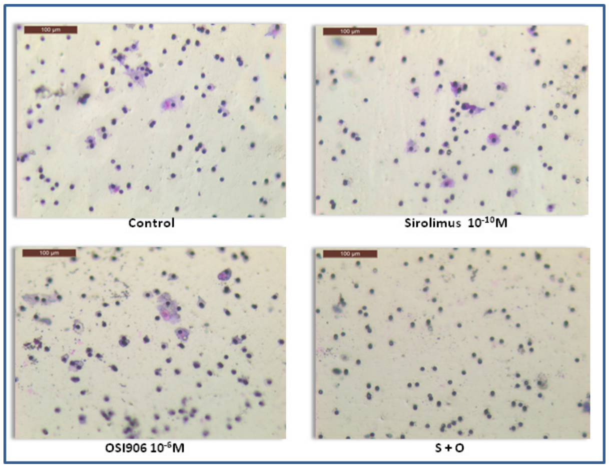

Supplementary Figure S3: Effects of Sirolimus and OSI-906, alone and in combination, on cell invasion after 24 hrs of treatment in HepG2 cell line. Since both drugs were diluted in DMSO, a double amount of DMSO was added to the control wells and the cells treated with the single agents were supplemented with an extra amount of DMSO.

Supplementary Table S1: Messenger levels of mTOR and IGF pathway components normalized against the expression of the housekeeping gene HPRT

	HepG2		HUH-7		
	mean	±S.E.M	mean	±S.E.M	p
IGF1	N.D.		N.D.		
IGF2	18,68	2,25	68,77	4,19	0,2
IGF1R	0,03	0,01	0,57	0,05	0,4
IGF2R	0,57	0,05	0,68	0,31	0,7
IRA	0,15	0,04	0,25	0,16	1
IRB	0,13	0,04	0,59	0,16	0,2
mTOR	0,19	0,07	0,66	0,14	0,11
pp70S6K	0,13	0,02	0,27	0,07	0,11
4eBP1	1,57	0,47	10,06	3,4	**0,02**

Supplementary Table S2: Percentage of inhibition, with p value vs control, and IC50 after 3, 6 and 9 days of treatment with OSI-906 and mTORi in both cell lines

		3 days			6 days			9 days		
		Min eff (%) p vs Ctr	Max eff (%) p vs Ctr	IC50	Min eff (%) p vs Ctr	Max eff (%) p vs Ctr	IC50	Min eff (%) p vs Ctr	Max eff (%) p vs Ctr	IC50
HepG2	OSI-906	1,7 ns	15,5 $p<0,05$	$9,9*10^{-8}$M	6,2 ns	21,4 $p<0,05$	$5,8*10^{-7}$M	6,3 ns	29,4 $p<0,001$	$2,5*10^{-7}$M
	Sirolimus	11,3 $p<0,05$	39,8 $p<0,001$	$8,8*10^{-12}$M	20,5 $p<0,01$	83,4 $p<0,001$	$1,6*10^{-11}$M	19,6 $p<0,01$	93,1 $p<0,001$	$1,7*10^{-12}$M
	Everolimus	4,6 ns	56,7 $p<0,001$	$3,8*10^{-13}$M	25,7 $p<0,001$	8 $p<0,001$	$2,2*10^{-13}$M	32,5 $p<0,001$	91,1 $p<0,001$	$3,6*10^{-13}$M
	Temsirolimus	6,5 ns	40,5 $p<0,001$	$1,1*10^{-12}$M	6,8 ns	81 $p<0,001$	$1,1*10^{-10}$M	7,9 ns	91,3 $p<0,001$	$1,0*10^{-11}$M
		3 days			6 days			9 days		
		Min eff (%) p vs Ctr	Max eff (%) p vs Ctr	IC50	Min eff (%) p vs Ctr	Max eff (%) p vs Ctr	IC50	Min eff (%) p vs Ctr	Max eff (%) p vs Ctr	IC50
HuH-7	OSI-906	13,1 ns	23,3 $p<0,05$	$6,4*10^{-11}$M	1,7 ns	9,17 ns	$1,4*10^{-6}$M	3,8 ns	23,1 $p<0,01$	$3,2*10^{-7}$M
	Sirolimus	18,9 $p<0,001$	47,0 $p<0,001$	$5,4*10^{-13}$M	32,6 $p<0,001$	69,2 $p<0,001$	$1,9*10^{-12}$M	51,8 $p<0,001$	95,9 $p<0,001$	$3,4*10^{-13}$M
	Everolimus	1,7 ns	28,7 $p<0,001$	$1,3*10^{-12}$M	7,3 ns	64,9 $p<0,001$	$4,1*10^{-12}$M	7 ns	83,3 $p<0,001$	$8,2*10^{-13}$M
	Temsirolimus	6,3 ns	42,2 $p<0,001$	$6,7*10^{-12}$M	11,7 ns	70,3 $p<0,001$	$1*10^{-11}$M	8,9 ns	95,8 $p<0,001$	$4,6*10^{-12}$M

Supplementary Table S3: Primers and probes for RT-qPCR. The sequences are 5′ - ′3 in FAM/TAMRA. Primers-probes were used in the following concentrations: HPRT 500-500-100 nM, IRA, mTOR and 4eBP1 300-300-100, IGF1, IGF2, IGF1R, IGF2R, IRB and p70S6K 300-300-200 of forward primer, reverse primer and probe, respectively.

Oligo Name	Forward	Reverse	Probe	Efficiency
IGF1R	CCAAAACTGAAGCCGAGAAG	GGGTCGGTGATGTTGTAGGT	AAGCAGGAACACCACGGCCG	1.85
IGF2R	ACCGACCCCTCCACGC	CCTCCAAGGCCACCTTCAG	AGCAGTACGACCTCTCCAGTCTGGCAAA	1.87
IGF1	TTGTGATTTCTTGAAGGTGAAGATG	CGTGGCAGAGCTGGTGAAG	TACCTGGCGCTGTGCCTGCTCA	1.98
IGF2	CCAAGTCCGAGAGGGACGT	TTGGAAGAACTTGCCCACG	ACCGTGCTTCCGGACAACTTCCC	1.98
IRA	CGTTTGAGGATTACCTGCACAA	GCCAAGGGACCTGCGTTT	TGGTTTTCGTCCCCAGGCCATC	1.89
IRB	CCCAGAAAAACCTCTTCAGGC	GGACCTGCGTTTCCGAGA	CTGGTGCCGAGGACCCTAGGCC	1.91
mTOR	TGCTGCGTGTCTTCATGCAT	GGATTGCAGCCAGTAACTTGATAG	ACAGCCCAGGCCGCATTGTC	1.91
p70S6K	TGGAAGACACTGCCTGCTTTT	TGATCCCCTTTTGATGTAAATGC	CTTGGCAGAAATCTCCATGGCTTTGG	1.86
4eBP1	GGCGGCACGCTCTTCA	TCAGGAATTTCCGGTCATAGATG	ACCACCCCGGGAGGTACCAGGA	1.94
HPRT	TGCTTTCCTTGGTCAGGCAGTAT	AAATCCAACAAAGTCTGGCTTATATC	CAAGCTTGCGACCTTGACCATCTTTGGA	1.91
